# A retrospective observational cohort study evaluating the postoperative outcomes of intracapsular coblation tonsillectomy in children

**DOI:** 10.1038/s41598-022-25768-0

**Published:** 2022-12-07

**Authors:** Mohamad A. Bitar, Tanvir Nazir, Hani Abd-Ul-Salam

**Affiliations:** 1 Division of Otolaryngology-Head and Neck Surgery, Al Jalila Children’s Specialty Hospital, Dubai, United Arab Emirates; 2 Mohammed Bin Rashid University of Medicine and Health Sciences, Dubai, United Arab Emirates; 3 Faculty of Dental Medicine and Oral Health Sciences, McGill University, Montreal, Canada; 4 Faculty of Dentistry, Gulf Medical University, Ajman, United Arab Emirates; 5 Faculty of Dentistry, University of Toronto, Toronto, Canada

**Keywords:** Outcomes research, Paediatric research, Quality of life

## Abstract

Coblation intracapsular tonsillectomy (ICT) is becoming popular due to its decreased postoperative complications. However, a concern exists about the need for revision surgery. We conducted a retrospective observational cohort study, with a null hypothesis that Coblation ICT is not associated with recurrence of the preoperative symptoms, obstructive tonsillar regrowth, or the need for revision tonsillar surgery. We reviewed 345 patients (median age of 4.5 years; IQR 3.2–6.3), operated by the senior author between Feb 2017 and Sep 2020, for a median follow-up of 395.0 days (IQR 221.5–654.5). Most patients had snoring (94.2%), mouth breathing (92.8%), restless sleep (62.6%), and sleep disorder breathing (52.8%); 12.5% had recurrent tonsillitis. The mean initial total symptoms score (TSS) was 5.2 (SD 1.4, range 1–8); 87.5% had three or more symptoms; 86.7% underwent ICT; TSS decreased postoperatively to a mean of 0.2, SD 0.8, range 0–7. The mean hospital stay was 0.96 day (SD 0.36, range 0–3). Secondary bleeding occurred in 0.7% of ICT patients. No patient required admission or intervention. There was no documented tonsillar regrowth resulting in upper airway obstruction. No one needed tonsillar revision surgery. Intracapsular tonsillectomy was shown to be an effective procedure with long-lasting results.

## Introduction

Adenotonsillar surgery is one of the most commonly performed surgeries in children. Many tools have been used to achieve this surgery with variable outcomes. The concept of intracapsular tonsillectomy (ICT) remerged after the work of Koltai using the microdebrider^1^. The National Prospective Tonsillectomy Audit (NPTA) assessed 33,921 extracapsular tonsillectomies (ECT), both adults and children, between 2003 and 2004. These constituted 70% of the total number of tonsillectomies in the United Kingdom during the same period. They reported a readmission rate of 3.9%, an incidence of post-operative hemorrhage of 3.5%, and a need to return to theatre in 0.9%^2^.

Though the risk of mortality post tonsillectomy is relatively low and estimated at 1/7132 to 1/170,000^3^, more concern was raised after the deaths of five young children who underwent ECT in Austria between 2006 and 2007. That resulted in a progressive switch to ICT in many places^4^.

The use of microdebrider for ICT became popular after the year 2002 due to the decrease in the degree of postoperative pain, quicker recovery, and lower rate of postoperative bleeding^1,5^. It was also found effective in improving the parameters of polysomnography in children^6^. The only concern was the excessive intraoperative bleeding, which was shown to increase two folds with the use of the microdebrider, though that was insignificant from a hemodynamic perspective^7^. Nevertheless, a shift towards using the Coblator has been noted mainly to avoid excessive intraoperative blood loss, something that the senior author also adopted.

Many studies have already been published on ICT (though the term was used interchangeably with tonsillotomy and tonsillar reduction, raising doubts on the amount of tonsillar tissues removed during the surgery), including using Coblation. Some focused only on the postoperative complications and recovery^8–10^, while others reported the outcome of such procedure^11–14^. In a metanalysis looking at the efficacy and complications of tonsillectomy versus tonsillotomy, the recurrence rate was found to be more common in the tonsillotomy group; however, various surgical tools were used in the analyzed studies, and it was not clear how much tonsillar tissues were removed during the procedures^11^. Similar findings were reported in a large cohort study, but various unspecified surgical instruments were used again, and multiple surgeons in different centers performed the surgeries^12^. A recent retrospective observational cohort study looking only at Coblation ICT in children younger than 16 years of age reconfirmed the lower complications rate. It could track the revision rate up to 5 years to be 2.2%. However, multiple centers and surgeons were involved, and the characteristic of the patients, including any existing comorbidities, were not analyzed^15^.

To evaluate the risk of recurrence while avoiding the confounding variables noted in the previous studies, we conducted a retrospective observational cohort study that included only pediatric patients that underwent tonsillar surgery using the Coblator, performed by a single surgeon, at a single center. Our null hypothesis was that Coblation ICT is not associated with the recurrence of the preoperative symptoms, obstructive tonsillar regrowth, or the need for revision tonsillar surgery.

## Methods

The study was approved by the Institutional Review Board of the Mohammed bin Rashid University of Medicine and Health Sciences in Dubai, UAE (study # AJCH-078; approval date 1 Mar 2021). The research was performed per the relevant guidelines and regulations for conducting research in Dubai healthcare city^16^. Informed consent was obtained from the participants’ legal guardians during the clinic visit before performing the surgery. Confidentiality was maintained all through the study. Consecutive patients undergoing tonsillar surgery using the Coblator between February 2017 and September 2020 were included in the study. Inclusion criteria included: children aged six months to 18 years, both males and females, surgery performed using the Coblator, ECT, or ICT, upper airway obstruction, recurrent tonsillitis (defined as seven episodes/year over one year, or five episodes/year for two years or three episodes/year over three years)^17^, and surgery performed by the senior author. Exclusion criteria included patients with craniosynostosis, previous tonsillar surgery, and patients who were lost to follow-up and could not be reached by phone.

The demographic data (age, gender) was reviewed. To analyze age as a potential variable, the patients were divided into three age groups: younger than 3 years of age, 3 to 6 years of age, and seven years and older. The preoperative and postoperative symptoms were noted, and the total number of symptoms was used for comparison and was labeled as the total symptoms score (TSS). The components of the TSS were snoring, mouth breathing, restless sleep, waking up at night, sleep disorder breathing, sore throat, dysphagia, runny nose, recurrent upper respiratory tract infection, and drooling. The TSS was calculated by adding the existing symptoms at each stage of the assessment (i.e., preoperatively, the first, and the last follow-up); for example, TSS 3 meant that there were three existing symptoms from the above list.

The degree of obstruction caused by the tonsils was classified according to Friedman’s grading scale^18^. Grade 0 signified that the patient had a previous tonsillectomy; grade 1 meant that the tonsils were barely seen behind the anterior pillars; grade 2 described the tonsils being visible behind the anterior pillars; grade 3 pointed to the tonsils extending three-quarters of the way to the midline; and grade 4 meant kissing tonsils. The tonsils were graded preoperatively in the clinic, intraoperatively on the surgical table, and during the postoperative follow-up visits. The comparison was made between the preoperative and intraoperative assessment of the tonsils to check the accuracy of examination in the clinic since not all children are cooperative for an adequate physical examination.

The preoperative assessment of the adenoids was done using lateral nasopharyngeal roentgenograms. The degree of obstruction was assessed at the level of the palatal airway^19^. The palatal airway was defined as the narrowest distance between the adenoid tissue and the soft palate. The degree of obstruction was estimated as grade 1 if less than 50% of the air column was obstructed; grade 2 if 50–75% obstruction was noted; grade 3 if an obstruction was more than 75%; and grade 4 if there was a complete obliteration of the air column. Intraoperative assessment of the adenoids was done using indirect nasopharyngoscopy using a laryngeal mirror and following the grading system mentioned above. The preoperative evaluation of the adenoids was compared to the intraoperative examination to evaluate the accuracy of palatal airway radiological interpretation.

Patients were admitted postoperatively if they lived more than half an hour from any medical facility, were younger than three years of age, had comorbidity (e.g., Down syndrome), had documented obstructive sleep apnea (OSA), or could not tolerate oral intake before discharge. All patients received intravenous hydration, antibiotics (cefazolin), and paracetamol (when needed for pain). Postoperative complications were documented during the first post-operative visit; we looked at postoperative bleeding (primary or secondary), dehydration, severe pain, respiratory distress, the need for readmission, and control of bleeding.

All patients were asked to follow up routinely at one month postoperatively, then at one year to assess any recurrence of symptoms. Those who failed to attend the extended follow-up were contacted by phone. We monitored any persistence or recurrence of symptoms and the need for revision surgery.

### Operation

The equipment we used for our tonsillar surgeries utilized a well-known technology delivered via plasma wands (Coblation wands; Arthrocare, a subsidiary of Smith & Nephew, Austin, TX, USA) and has been well described in the company product’s documents and website^20^. The setup that we used for all our cases will be described below as well as the selection of the wands depending on the preference and the experience of the senior author (MAB).

Under general anesthesia, with the patient in the Rose position, the mouth was opened using a mouth gag (McIvor mouth gag; Rudolf, Fridingen, Germany). Two 8 Fr catheters (Dover Nelaton urinary catheter; Covidien, Mansfield, MA, USA) were introduced through the nasal cavities to retract the soft palate. A laryngeal mirror (laryngeal mirror; Karl Storz, Tuttlingen, Germany) was used to visualize the adenoid pad. This was sometimes coupled with a 30-degree, 18 cm long, 2.7 mm diameter telescope (Hopkins II forward-oblique telescope 30°; Karl Storz, Tuttlingen, Germany) when the patient was very young with limited transoral access or when the adenoids extended inside the posterior choanae.

The ablation of the adenoids was performed from inferior to superior, ensuring keeping the lower edge of the adenoids intact to provide an adequate ridge in the Passavant sphincter area to avoid the postoperative development of velopharyngeal insufficiency. Care was taken to remove all obstructive tissues and ensure clearance of both the posterior choanae and the Eustachian tubes. Hemostasis was achieved using the coagulation mode of the Coblator. The type of wand used for the adenoids depended on the choice for the tonsillar surgery. The default setting of the Coblator was often used, but the ablation power was occasionally increased to 8 or 9 in case the adenoid tissue was dense (usually in older children). Both the adenoids and tonsillar surgeries were performed under magnification. That was initially provided by a surgical loupe (front-lens-mounted 2.5 × magnification loupes; Surgitel, Ann Arbor, MI, USA); however, with the emergence of the covid pandemic, we shifted to the microscope (OPMI Sensera; Zeiss, Oberkochen, Germany) that provided a mean to keep the surgeon away from the oral cavity. This practice has become the norm till the present time.

### Intracapsular tonsillectomy

The senior surgeon (MAB) chose the type of wands as follows: a small wand (PROCISE EZ View wand; Arthrocare, a subsidiary of Smith & Nephew,  Austin, TX, USA) was used for children younger than two years of age; a medium wand (EVAC 70 HP; Arthrocare, a subsidiary of Smith & Nephew, Austin, TX, USA) was used for children 2–5 years of age and a large wand (PROCISE MAX; Arthrocare, a subsidiary of Smith & Nephew, Austin, TX, USA) was used for children older than five years of age. This choice was not rigid and was sometimes influenced by the size of the tonsils, the size of the oral cavity, and the existing space that allowed adequate movement of the wand inside the mouth. The Coblation was carried on from the most protruding part of the tonsil and progressed along its length from anterior to posterior, avoiding working in blind deep craters.

A pillar retractor (Hurd dissector and pillar retractor, Karl Storz, Tuttlingen, Germany) was used to protect the surrounding soft tissues (uvula, soft palate, posterior pillar, base of tongue, and posterior pharyngeal wall) and to expose the tonsillar tissues in different parts of the tonsillar bed. Care was taken to address the superior pole, where tissues could be easily missed. Visualization of this area was made possible by using a laryngeal mirror (laryngeal mirror; Karl Storz, Tuttlingen, Germany).

Coblation was carried on until all the crypts were flattened. If the indication was purely obstructive, reaching the capsule was unnecessary, especially if the tonsil was entirely embedded or the child was younger than two years of age (parents are often concerned about immunity and usually ask to leave tonsillar tissues in place). Otherwise, the tonsillar tissues were Coblated until the capsule was identified by its greyish color. Care was taken to Coblate and sometimes coagulate (denature) the islands of tonsillar tissue left behind. Hemostasis was achieved using the coagulation mode of the Coblator. The default setting of the Coblator (7 ablation, 4 coagulation) was usually used; however, a higher ablation setting was occasionally used (8 or 9) when the tonsillar tissues were dense or the tonsil was massive.

### Extracapsular tonsillectomy

The preferred tool was a small wand with a flat plate (PROCISE EZ; Arthrocare, a subsidiary of Smith & Nephew, Austin, TX, USA), which was ideal for a clean dissection between the tonsillar capsule and the bed’s muscles. It was the preference of the senior author to use a lower ablation power of 4. Any encountered vessel or bleeder was addressed using the coagulation mode at the default setting of 4.

### Statistical analysis

As this procedure is already known to be effective^12–14^, a difference between the postoperative and perioperative mean TTS of at least 1.0 would be expected. With a power of 0.9 and alpha 0.05, only 11 patients were needed to show this improvement. However, this study aimed to look at the persistence of this improvement over time. So we included all operated patients during the specified period to increase the chance of detecting any recurrence and thus the need for revision surgery.

Statistical analyses were performed using statistical software (Statistical Package for the Social Sciences, SPSS Statistics for Windows, Version 22.0; IBM, Armonk, NY, USA). Descriptive data were expressed as mean ± SD and range, for continuous measures or as a percentage for discrete measures. Median and interquartile range (IQR) were used for non-parametric data. A kappa statistic measure of agreement was performed to evaluate the pre-operative and intra-operative tonsillar and adenoidal grades and was presented as a percentage. A level of agreement below 40% was considered low, between 40 and 70% as moderate, and above 70% as high. Spearman's rank correlation and X^2^ tests were performed to measure the degree of association between variables. A significance level α of less than 0.05 was used.

## Results

**Figure 1:**
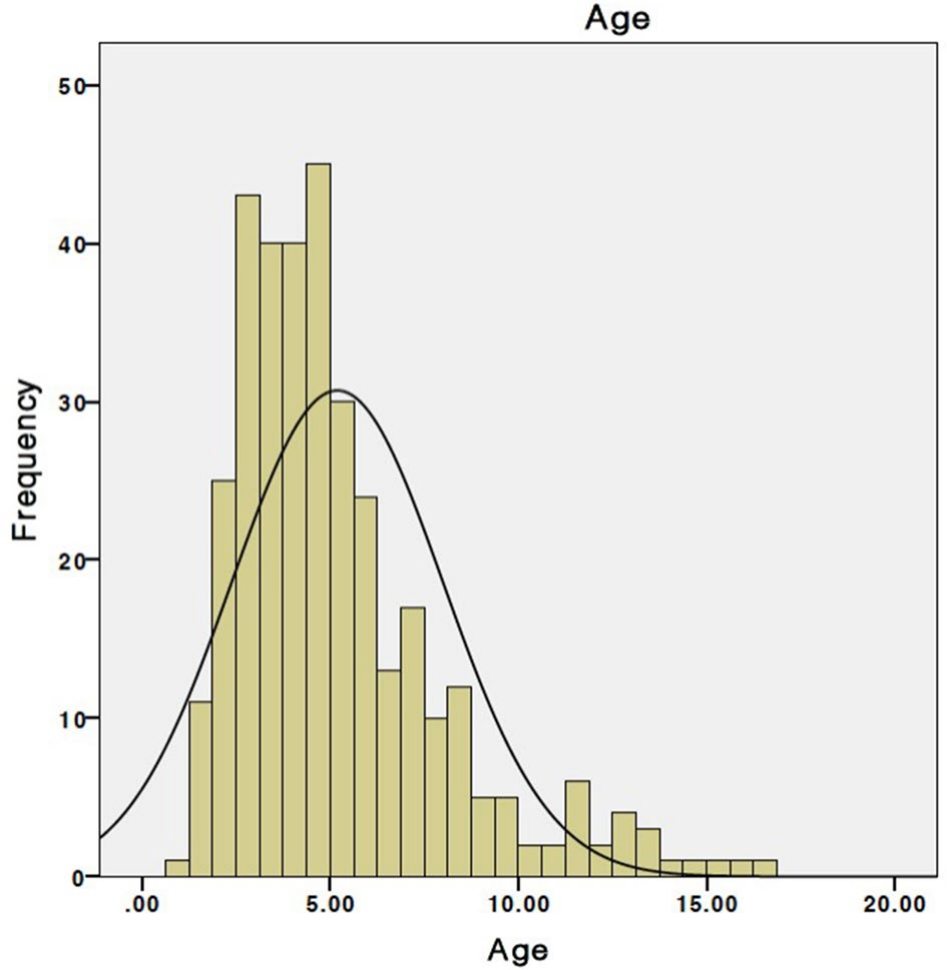
Age (years) distribution of the studied patients.

**Figure 2:**
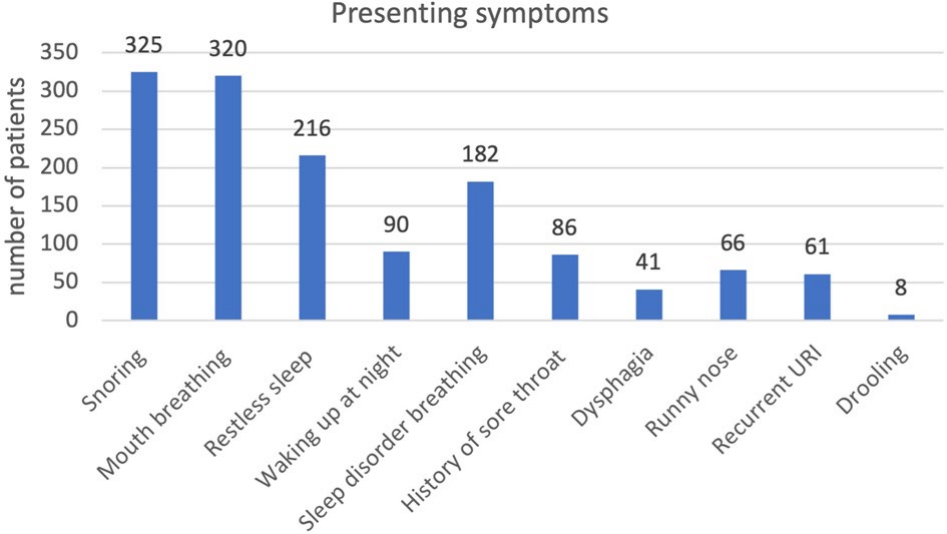
Presenting symptoms of the children undergoing Coblation-assisted adenotonsillar surgery.

We reviewed 345 patients, 208 of them were male (1.5/1 = M/F), with a median age of 4.5 years, IQR 3.2–6.3; 79.7% of them were younger than seven years (Fig. 1). The patients presented mainly with upper airway obstructive symptoms; the various symptoms were summarized in Fig. 2. Only 39 patients had polysomnography; with 33.3% having severe, 43.6% moderate and 23.1% mild OSA.

**Figure 3:**
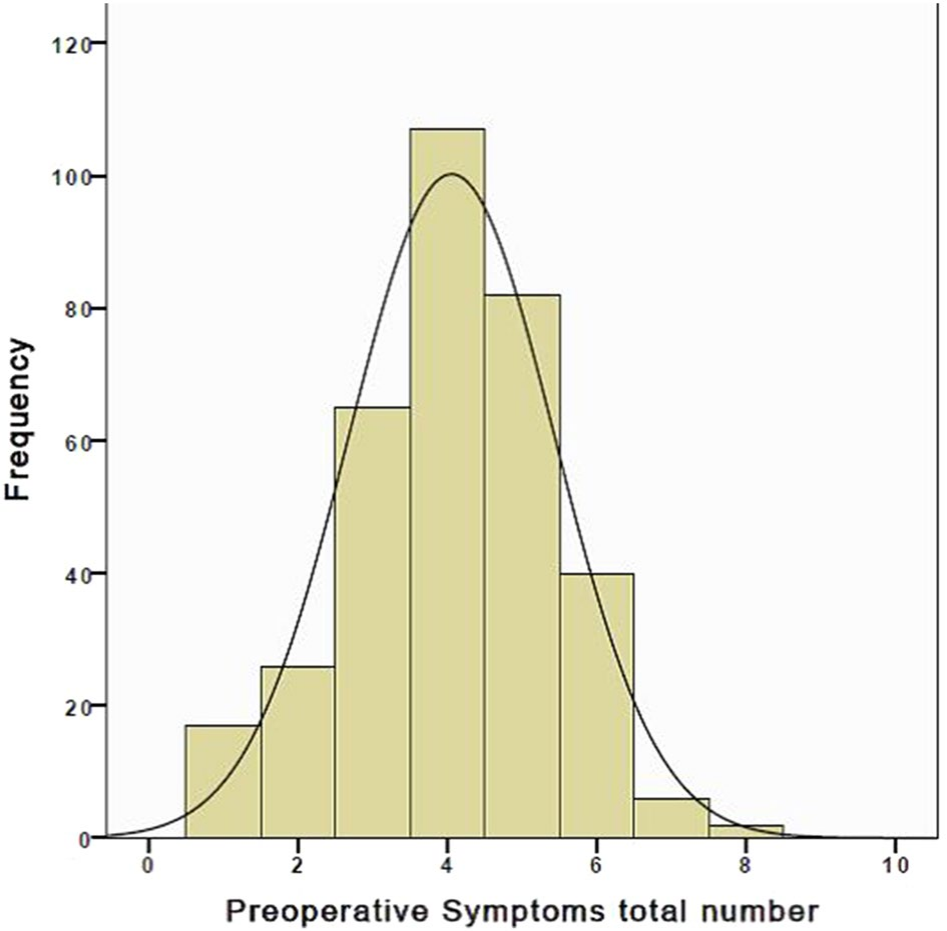
Preoperative total symptoms score (TTS).

The patients had a preoperative mean TSS of 5.2 (SD 1.4; range 1–8), with 87.5% having three or more symptoms at the time of presentation (Fig. 3). There was a significant negative correlation between the age of the patient and the degree of preoperative symptoms (*P* < 0.001). The results of the comparison between the preoperative and intraoperative assessments of the tonsils and adenoids are detailed in Table 1. There was moderate agreement between the preoperative and intraoperative evaluation of the tonsils (Kappa 0.433). The agreement between the preoperative and intraoperative assessment of the degree of obstruction caused by the adenoids was low (Kappa 0.215). The postoperative tonsillar grades were summarized in Table 1 to document the changes in the degree of obstruction.

**Table 1:** Preoperative, intraoperative, and postoperative assessment of the tonsils; and preoperative and intraoperative assessment of the adenoids.

	G0 (No/%)	G1 (No/%)	G2 (No/%)	G3 (No/%)	G4 (No/%)
**Tonsillar size**					
Preoperative assessment	0/0	3/0.9	26/7.5	139/40.3	177/51.3
Intraoperative assessment	0/0	0/0	6/1.7	150/43.5	189/54.8
Postoperative assessment (first follow up)	42/12.2	298/86.4	4/1.1	1/0.3	0
Postoperative assessment (last follow up)	23/6.7	315/91.3	7/2.0	0	0
Adenoids’ degree of obstruction*					
Preoperative assessment	0	21/8.4	55/22.0	110/44.0	64/25.6
Intraoperative assessment	0	8/2.3	156/45.2	97/28.1	84/24.4

95 patients (27.5%) did not have an X-ray done.

*G* grade, *No* number of patients.

*Postoperative adenoids assessment was not done as routine as it required an X-ray or endoscopy, which were not indicated unless revision surgery was considered.

Most patients had adenotonsillar hypertrophy (87.0%) as an indication for surgery; the rest (13%) were operated on for mainly recurrent tonsillitis. Fifty-nine patients (17.1%) had comorbidities, such as Down syndrome, hypotonia, reactive airway disease, isolated cleft palate, cardiac problem, diabetes type 1, receiving growth hormone, autism, thalassemia, Familial Mediterranean Fever (FMF), and Pediatric Autoimmune Neuropsychiatric Disorders Associated with Streptococcal Infections (PANDAS).

Intracapsular tonsillectomy was performed in 86.7% of the studied children (299 patients); 9 did not have concomitant adenoidectomy. This was mostly done for an obstructive indication. On the other hand, ECT was performed mainly for recurrent tonsillitis (34/46 or 74.0%). The ICT and ECT groups' characteristics were summarized in Table 2.

**Table 2:** Comparison of characteristics of both intracapsular and extracapsular groups.

	Intracapsular tonsillectomy group	Extracapsular tonsillectomy group	*P* value
**Age**			0.001
Mean	4.9	6.8	
SD	2.6	3.3	
Range	11 months–16.3 years	3–15.7 years	
**Gender**			0.125
Male	185 (61.9%)	23 (50%)	
Female	114 (38.1%)	23 (50%)	
**Diagnosis**			0.146
ATH	288 (96.3%	12 (26.1%)	
RT	11 (3.7%)	34 (73.9%)	
**Preoperative TTS**			0.001
Mean	4.1	3.3	
SD	1.3	1.7	
Range	1–8	1–7	
**Preoperative tonsillar grading**			0.023
Mean	4	3	
SD	0.6	0.8	
Range	1–4	1–4	
**Intraoperative adenoidal grading**			0.004
Mean	3	2	
SD	0.9	0.7	
Range	1–4	1–4	

*SD* standard deviation, *ATH* adenotonsillar hypertrophy, *RT* recurrent tonsillitis, *TTS* total symptom score.

**Figure 4:**
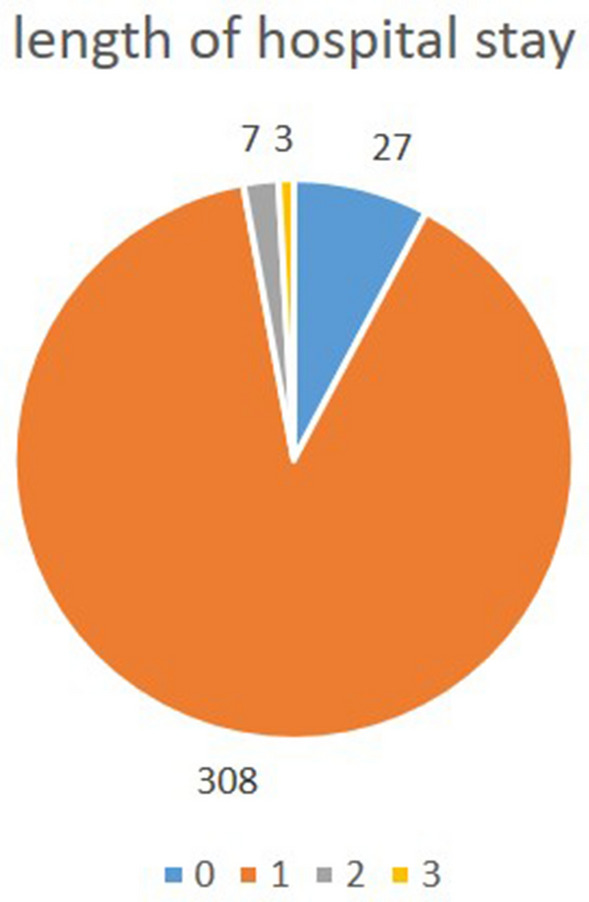
Duration of hospitalization in days.

The postoperative course was smooth and uneventful in most of the patients, with a mean stay in the hospital of 0.96 days (SD 0.36; range 0–3) (Fig. 4). No correlation was found between the age of the patients (*P* = 0.221), the preoperative TTS (*P* = 0.546), or the type of surgery (*P* = 0.156) and the length of stay in the hospital.

Postoperative complications included secondary bleeding that occurred in 1.7% of the patients [0.7% (2 patients) of the ICT group; 8.7% (4 patients) of the ECT group) and respiratory distress (0.3%) that occurred in one patient belonging to the ICT group. No correlation was found between the age group and the occurrence of postoperative bleeding (*P* = 0.805). None of the patients who had postoperative bleeding required admission to the hospital or control of bleeding in the operating room.

**Figure 5:**
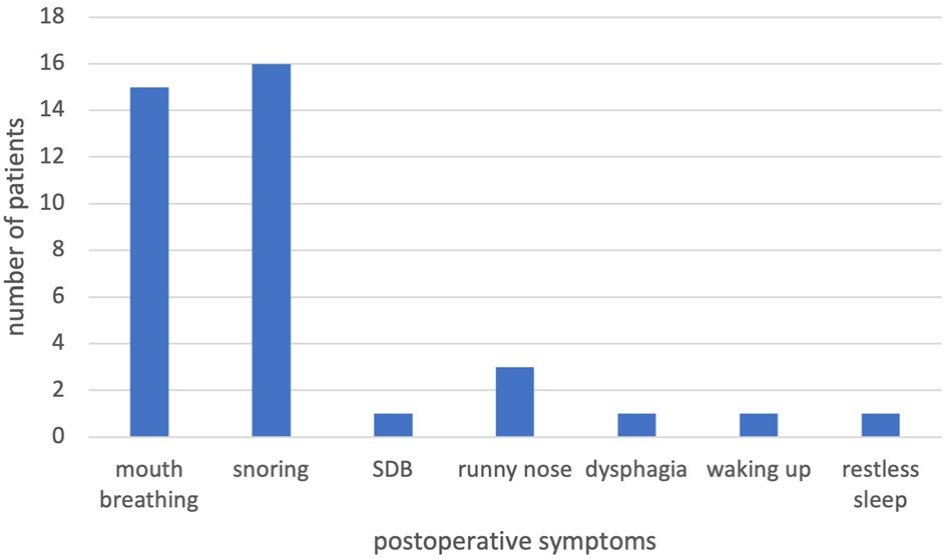
Persistent symptoms that were noted at the first postoperative follow up.

The first postoperative follow-up occurred at a median of 35.0 days, with IQR 29.0–49.75. Thirty-three patients (11.8%) had a median persistent TSS score of 0.0, IQR 0.0–0.0; 88.2% had no symptoms, 7.6% had one symptom, 2.1% had two symptoms, and 2.1% had three or more symptoms (Fig. 5). All patients with residual symptoms had ICT; 21.2% of them had preoperatively documented OSA, and 15% had comorbidities. The last postoperative follow-up occurred at a median of 395.0 days, IQR 221.5–654.5. Only nine patients had residual (1-3) symptoms (Table 3); one needed a revision adenoidectomy.

**Table 3:** Distribution of the TSS among our patients preoperatively and at first and last follow-up visits.

No of symptoms	Preoperative TSS	Postoperative TSS (First follow-up)	Postoperative TSS (Last follow-up)
	No of patients/percentage	No of patients/percentage	No of patients/percentage
0	0/0	312/90.4	336/97.4
1	17/4.9	21/6.1	5/1.4
2	26/7.5	6/1.7	3/0.9
3	65/18.8	1/0.3	1/0.3
4	107/31.0	2/0.6	0/0
5	82/23.8	1/0.3	0/0
6	40/11.6	1/0.3	0/0
7	6/1.8	1/0.3	0/0
8	2/0.6	0/0	0/0

*TSS* total symptoms score.

There was no correlation between age and the persistence of postoperative symptoms, whether at the first follow-up (*P* = 0.951) or the last follow-up (*P* = 0.978). No patient had recurrent tonsillitis postoperatively at the first and last follow-up.

## Discussion

Our study showed that Coblation ICT was not associated with the recurrence of the preoperative symptoms, obstructive tonsillar regrowth, or the need for revision tonsillar surgery.

Intracapsular tonsillectomy has been progressively gaining its place as an alternative to ECT. The advantages of ICT in decreasing postoperative pain and providing a faster return to regular diet have been previously reported, initially using the microdebrider^1,5^. The microdebrider has also shown effectiveness in improving OSA symptoms in children^6,21^. However, the presence of unnecessary intraoperative blood loss during the procedure, and the difficulty in safely reaching the capsule without the risk of violating it, made some surgeons prefer more hemostatic tools like lasers, radiofrequency, and Coblation^7^. In a prospective, randomized study, these three techniques were compared regarding postoperative recovery and recurrence rate^22^. The pain was the highest among the laser group, while analgesic usage, return to a regular diet, and resuming routine activity were the lowest in the Coblator group. In addition, none of the Coblator cases needed reoperation (vs. 21.4% in the radiofrequency and 8.3% in the laser groups).

Another main drive to switch from ECT to ICT was the significant decrease in postoperative bleeding rate. In a retrospective cohort study comparing the postoperative course of ICT vs. ECT, a significant drop was found in the postoperative bleeding rate (0.48% in ICT vs. 3.61% in ECT)^9^. In a more recent retrospective study reviewing 730 patients, both adults and pediatrics, the postoperative bleeding was compared between ICT and ECT using the Coblator. Again, a significant decrease in the rate of bleeding was observed in the ICT group (0.3% vs. 2.1%; *P* = 0.025)^23^.

In other studies that looked prospectively at the outcome of Coblation ICT, the prevalence of postoperative bleeding was again found to be significantly lower (0.4–0.5%) than the well-known rate of post-tonsillectomy bleeding (3.5%)^2,13,14^. Some even reported no postoperative bleeding at all^10^. Our experience with Coblation ICT confirmed a similar drop in the postoperative bleeding rate, which was mild, brief, and self-limiting. This is a significant improvement in the safety of tonsillar surgery.

The relation of age to the risk of post-tonsillectomy bleeding is well known in the literature, showing a persistent higher incidence in adults compared to children^2^, whether undergoing ECT or ICT^23^. Looking solely at the pediatric age group, it was previously reported that the older the child, the higher the risk of postoperative bleeding; 1.9% for children < 5 yo, 3.0% for children 5–15 yo, and 4.9% for 16 yo and older^2^, but that was in patients who underwent ECT. As the incidence of postoperative bleeding is almost ten folds less in children undergoing ICT^9^, a correlation with the various age stratifications within the pediatric population might be hard to demonstrate, something we encountered in our study.

The ICT was initially introduced to address the tonsils and adenoids causing upper airway obstruction in children with sleep-disordered breathing. Though it was proven effective in relieving these patients' symptoms, there were concerns about the possibility of recurrence of symptoms in the long run^11^ or its association with residual OSA documented on repeat polysomnography (PSG)^21^. However, it is known that this issue is not specific to ICT, as residual OSA has been previously reported following ECT, especially in children younger than three years of age, with the severity of preoperative OSA being a positive predictor factor^24^. In a retrospective cohort study looking at the clinical outcomes of both ICT and ECT in a group of children with PSG-confirmed OSA, it was noticed that some patients with comorbidities like asthma and obesity had residual OSA on repeat PSG in the ICT group^21^. These findings highlight the importance of adequately counseling the parents regarding each technique’s advantages and disadvantages and considering the comorbidities existing in the patient.

The persistence of some preoperative symptoms or their recurrence later does not necessarily mean the need for a new surgical intervention. We have looked at our patients who had residual symptoms postoperatively and noticed that either they had comorbidities or severe preoperative obstructive symptoms. Most of the patients could be managed medically. A recent large series of children undergoing Coblation ICT found that being younger than two years of age, having severe OSA, or suffering from severe comorbidities were risk factors for revision surgery. In general, the parents must be informed of the possible need for a revision adenotonsillar surgery in 2.4–2.6% of all patients^13,14^, though this rate has not been universally reported.

The preoperative assessment is essential when deciding on the need for adenotonsillar surgery. We noticed that the tonsillar evaluation on the physical examination in the clinic could underestimate the degree of obstruction caused by the tonsils due to the difficulty in examining the throat of children. Therefore, it is essential to correlate the examination findings with the presenting symptoms to decide on the best surgical option. In addition, the adenoidal assessment by lateral nasopharyngeal X-ray showed that the degree of obstruction may only sometimes be accurate with some margin of error when estimating grade 2 and grade 3 compared to the intraoperative findings. Clinical correlation is again of great importance.

As ICT became a more common technique among surgeons, its application spread beyond sleep-disordered breathing to include infectious indications. In a series of 500 patients undergoing ICT, infective and obstructive indications were included in the selected candidates^13^. Their positive outcome challenged the concept of avoiding ICT in patients with a history of recurrent sore throat. This report encouraged us to include these patients as candidates for ICT. The rationale for its expected efficacy in tonsillitis is that ICT eliminates the crypts and most lymphoid tissue in the tonsillar fossa. Even when performing ECT for recurrent tonsillitis, sore throat cannot be totally prevented. It does decrease the frequency of sore throat for at least a year post-operatively, but there is not enough evidence that it will avoid it in the long run^25^.

We have noticed that using magnification, reaching the capsule during ICT was done more effectively and safely. Though that could be achieved with the surgical loops, we have found the microscope to be more helpful in providing fine details when ablating the tonsillar tissue islands at the end of the procedure. Using the microscope was also beneficial in broadcasting the surgical procedure during teaching and training sessions. The use of magnification (namely the microscope) was previously evaluated in a retrospective study that showed its correlation with a decrease in the duration of hospital stay, a drop in the incidence of postoperative secondary hemorrhage, and improvement in postoperative pain scores^26^.

As with any new technology, the cost is always an issue, which can make it unavailable in low-income countries. However, the benefits can sometimes outweigh the costs, making it more cost-effective in the long run. In a recent study that compared the cost–benefit of using Coblation versus electrocautery in ECT in children, it was found that performing the surgery using electrocautery carried a significant increase in indirect costs. These were related to increased operative time, more extended stay in PACU, and more need for pain killers^27^. If we consider Coblation ICT, we can add the significant decrease in the risk of postoperative bleeding, which will further contribute to the reduction in the overall cost of the procedure.

Limitations of this retrospective observational cohort study include the absence of a control group in which another surgical tool was used. However, that was not possible as Coblation was our only used technology for tonsillar surgeries. Another limitation was the small number of patients in the ECT group, which limited the comparison between ICT and ECT. However, that could not be avoided as the decision to undergo the specific type of procedure was that of the caregivers. We were also in a transition period to switch from ECT to ICT for most of our tonsillar surgeries. That impacted the prevalence of the postoperative bleeding rate in the ECT group, which was much higher than the one reported in the literature (8.7% vs. 3.5%). The rate could have been different if more patients were present in that group. An additional limitation is related to the duration of follow-up, though we believe it was acceptable based on previously published large series^13^. In that report, a mean follow-up of 7.4 months revealed a 2.4% recurrence rate. The same group published more recent results with longer follow-ups (mean of 46.6 months) which showed almost the same recurrence rate (2.7%)^14^, which gives an impression that the recurrence rate does not seem to worsen over time.

## Conclusion

Coblation ICT was found to be an effective procedure in children with upper airway obstruction and recurrent tonsillitis. No recurrence of preoperative symptoms was seen during the documented follow-up period. No tonsillar regrowth causing upper airway obstruction was noted, nor there was a need for revision tonsillar surgery, alleviating these concerns that have been often raised.

## Data Availability

The data sets generated and analyzed during the current study are not publicly available due to medical confidentiality. Still, they are available from the corresponding author on reasonable request in a summarised form upon approval from the IRB.
